# Clinical Approach to the Standardization of Oriental Medical Diagnostic Pattern Identification in Stroke Patients

**DOI:** 10.1155/2011/768492

**Published:** 2010-09-19

**Authors:** Han Jung Kim, Hyung Sup Bae, Seong Uk Park, Sang Kwan Moon, Jung Mi Park, Woo Sang Jung

**Affiliations:** Department of Cardiovascular & Neurologic Diseases (Stroke Center), College of Oriental Medicine, Kyung-Hee University, Seoul # 130-702, Republic of Korea

## Abstract

In Korea, many stroke patients receive oriental medical care, in which pattern-identification plays a major role. Pattern-identification is Oriental Medicine's unique diagnostic system. This study attempted to standardize oriental medical pattern-identification for stroke patients. This was a community-based multicenter study that enrolled stroke patients within 30 days after their ictus. We assessed the patients' general characteristics and symptoms related to pattern-identification. Each patient's pattern was determined when two doctors had the same opinion. To determine which variables affect the pattern-identification, binary logistic regression analysis was used with the backward method. A total of 806 stroke patients were enrolled. Among 480 patients who were identified as having a certain pattern, 100 patients exhibited the Fire Heat Pattern, 210 patients the Phlegm Dampness Pattern, nine patients the Blood Stasis Pattern, 110 patients the Qi Deficiency Pattern, and 51 patients the Yin Deficiency Pattern. After the regression analysis, the predictive logistic equations for the Fire Heat, Phlegm Dampness, Qi Deficiency, and Yin Deficiency patterns were determined. The Blood Stasis Pattern was omitted because the sample size was too small. Predictive logistic equations were suggested for four of the patterns. These criteria would be useful in determining each stroke patient's pattern in clinics. However, further studies with large samples are necessary to validate and confirm these criteria.

## 1. Introduction

The prevalence rate of stroke has been rising recently, as the average life span has become longer. Since stroke can be fatal at acute stages and may leave irreversible neurological deficits, it has generated a great deal of social concern. In modern Western medicine, thrombolysis and anticoagulants are conventionally used. However, these are insufficient and are known to pose some adverse effects including a hemorrhagic tendency [1–3]. Therefore, alternative medical practices have attracted considerable attention. In Korea especially, many stroke patients are receiving oriental medical care [4] because Korea has its own traditional alternative medicine called Traditional Korean Medicine (TKM) or Oriental Medicine (OM), the role of which has been emphasized in stroke management. OM is a comprehensive system of medicine characterized by its own theoretical basis and practical experience. Pattern-identification is its unique diagnostic system entailing comprehensive analysis of symptoms and signs with implications for determining the cause, nature, and location of the illness, the patient's physical condition, and the patient's treatment. These features can lead OM doctors to care for their patients based on individualized treatment, magnifying the effectiveness of OM and minimizing its adverse effects. 

There has been little progress, however, in the standardization of oriental medical pattern-identification. In Japan, several standardized questionnaires have been suggested and used in the literature. All these questionnaires, except for the diagnostic criteria for *OKETSU* made by Terasawa et al. [5], do not clearly state their methodology or how they were developed. In China, a standard form of pattern-identification for stroke patients has been announced [6]. The authors believe, however, that this form is impractical because it forces a doctor to choose only one pattern from among several patterns suggested in the form. Thus, mixed and combined patterns, which are frequently seen in clinics, cannot be identified with such method. In Korea, several questionnaires for pattern-Identification have been developed recently [7–10]. They were developed with reasonable statistical methods, but they cannot satisfy the objective of this study, because each of them is based on a very small sample size of less than 100 subjects. Furthermore, stroke was not taken into consideration. Thus, given the limitations of the aforementioned efforts, this study attempted to standardize the oriental medical pattern-identification for stroke patients.

## 2. Methods

### 2.1. Subjects

This study was a community-based multi-center trial. We enrolled stroke patients within 30 days after their ictus from the oriental medical university hospitals in the National Capital Region, including Seoul and Kyunggi-do. Kyung Hee Oriental Medical Center (Seoul), Kyung Hee East-West Neo Medical Center (Seoul), Dong Guk International Hospital (Kyunggi-do), and Kyung Won Oriental Medical Hospitals (Seoul and Incheon) were the involved hospitals. We excluded traumatic stroke such as subarachnoid, subdural, and epidural hemorrhage. Informed consent of all the study patients was obtained after a thorough explanation of the details. This study was approved by the Institutional Review Board (IRB) of Kyung Hee Oriental Medical Center (KOMC IRB 2007-07).

### 2.2. Measured Variables

In this study, we used the Case Report Form (CRF) and the Standard Operation Procedures (SOPs) developed by the Experts Committee organized by the Korean Institute of Oriental Medicine [11–14]. These included general patient information such as diagnosis, the Trial of ORG 10172 in Acute Stroke Treatment (TOAST) classification of the stroke [15, 16], medical history, and smoking habits. Table 1 shows the variables related to oriental medical pattern-identification. We translated the terms of oriental medicine into English based on the guideline suggested by the World Health Organization (WHO) [17].

### 2.3. Determination of Pattern-Identification

Two oriental internal medical doctors from each hospital and who had at least more than three years of clinical experience with stroke identified the pattern of each patient as follows the Fire Heat Pattern, the Phlegm Dampness Pattern, the Blood Stasis Pattern, the Qi Deficiency Pattern, and the Yin Deficiency Pattern, as suggested by the Korean Institute of Oriental Medicine [11–13]. In the very case, where two doctors had the same opinion, the pattern of the patient was confirmed.

### 2.4. Statistics

To determine which variables affect the pattern-identification, binary logistic regression was used. We removed the variables without statistical significance step by step using the backward method until a combination composed of purely significant variables was reached and considered each retained variable as an independent factor for the predictive model. The derived logistic equation was log (*P*/1 − *P*) = *β*_0_ + *β*_1_ *X*_1_ + *β*_2_ *X*_2_ + ⋯ +*β*_*n*_ *X*_*n*_, where the *X*s are independent variables, the *β*s are regression coefficients, and *P* is the probability of the pattern-identification. The entire analysis was performed using SPSS for Windows, version 12.0 (SPSS Inc., Chicago, Illinois, USA).

## 3. Results

### 3.1. General Characteristics of Each Pattern-Identification

A total of 806 stroke patients were recruited. Of these, 326 could not be classified as having a certain pattern because the diagnoses of the two doctors differed. These patients were excluded from the analyses. The diagnostic differences may be explained by the subjectivity of pattern-identification and the absence of objective standard criteria, that is, precisely why this study is necessary. Among the remaining 480 patients, 100 patients had the Fire Heat Pattern, 210 patients the Phlegm Dampness Pattern, 9 patients the Blood Stasis Pattern, 110 patients the Qi Deficiency Pattern, and 51 patients the Yin Deficiency Pattern. There were more male patients with the Fire Heat Pattern, and there were more patients who exhibited more variables related with the metabolic syndrome such as hypertension, diabetic mellitus, and hyperlipidemia under the Phlegm Dampness Pattern. As for TOAST classification [15, 16], all the patterns showed similar results, where small vessel occlusion (SVO) was the most common (Table 2). Only descriptive statistics were applied, however, because of the lack of balance in the sample sizes of the patterns. 

To come up with a predictive model for pattern-identification, binary logistic regression analysis was applied using the backward method on the assessed pattern-related variables. Fire Heat, Phlegm Dampness, Qi Deficiency, and Yin Deficiency were studied. The Blood Stasis Pattern was omitted because the sample size was too small.

### 3.2. The Retained Significant Symptoms for Each of Five Patterns

A total of 100 patients (20.8% of all the patients) were identified as having the Fire Heat Pattern. All of the assessed variables were included in the first logistic regression analysis. Among the statistically significant variables (*P* < .1) found, afternoon tidal fever, white fur, and slow pulse were less statistically significant and were removed earlier. Nausea, floating pulse, and deep pulse were found to have been relatively very significant in the first analysis. They were removed, however, because their significance decreased while the backward method progressed. Aversion to heat, pale tongue, and strong pulse had low statistical significance at first, but these increased as the backward method progressed. Reddened complexion, thin pulse, slippery pulse, eyeball congestion, thick fur, and teeth marked tongue had high significance levels and, thus, were retained.

Phlegm Dampness Pattern was identified in 210 patients (43.8% of all the patients). Thick fur and slow pulse had lower significance and were removed earlier from among the significant variables (*P* < .1) in the first analysis. The significance levels of spotted tongue, rapid pulse, deep pulse, eyeball dryness, and night sweating were relatively high at first, but the levels decreased as the backward method progressed Fatigue and pale tongue had higher significance levels after the other variables were removed. Overweight, pale complexion, reddened complexion, flushed cheeks, and slippery pulse had high significance levels and, thus, were retained.

A total of 110 patients (22.9% of all the patients) were identified as having the Qi Deficiency Pattern. Among the statistically significant variables (*P* < .1), variables with lower significance levels such as profuse sweating and afternoon tidal fever were removed earlier. Faint low voice, borborigmus, and thin pulse had high significance levels in the first analysis, but were removed as the backward method progressed. The significance levels of vacuous pulse and nausea were low at first, but strengthened and were retained. Overweight, pale complexion, reddened complexion, flushed cheeks, eyeball dryness, night sweating, reversal cold in the extremities, thick fur, deep pulse, rapid pulse, and slippery pulse had high significance levels and, thus, were retained.

There were 51 patients, or 10.6% of all the patients, with the Yin Deficiency Pattern. Among the statistically significant variables (*P* < .1), night sweating, heat in the palms and soles, mirror tongue, white fur, and rough pulse were removed earlier as the backward method progressed. Flushed cheeks, thirst, afternoon tidal fever, dry fur, rapid pulse, and strong pulse had high significance levels and, thus, were retained. So that the characteristics of each pattern may be seen as a whole, a figure of *β* regression coefficients is presented here.

## 4. Discussion

This is the first study to standardize oriental medical diagnostic pattern-identification of stroke patients in Korea. The following predictive logistic equation was set log (*P*/1 − *P*) = *β*_0_ + *β*_1_ *X*_1_ + *β*_2_ *X*_2_ + ⋯ +*β*_*n*_ *X*_*n*_, where the *X*s are independent variables, the *β*s are regression coefficients, and *P* is the probability of the Pattern-Identification. It is a popular method of building a predictive model that has a familiar interpretation. According to the *β* coefficient, there would be positively quantified or negatively quantified variables. This means that if a patient has more positives, the probability of the pattern-identification being correct would increase, while if a patient has more negatives, the probability of the pattern-identification being incorrect would increase.

The Fire Heat Pattern is by pathogenic Fire characterized by intense heat that is apt to injure fluid, consume Qi, engender wind, induce bleeding, and disturb mental activities [17]. In this study, the predictive logistic equation for the Fire Heat Pattern is as follows log   (*P*/1 − *P*) = 3.021*(Reddened complexion) + 1.052*(Eyeball congestion) + 0.682*(Aversion to heat) − 1.388*(Pale tongue) + 0.727*(Thick fur) − 1.134*(Teeth marked tongue) + 1.295*(Strong pulse) − 1.122 (Thin pulse) − 0.972*(Slippery pulse) − 2.865. Among the positively quantified variables, reddened complexion, eyeball congestion, and aversion to heat are traditionally considered the indicators for Fire or Heat in oriental medicine. Thick fur and strong pulse are the indicators of the excessive patterns to which the Fire Heat Pattern belongs. Thus, the above mentioned positive variables seem reasonable. With the negatively quantified variables, the Fire Heat Pattern could be distinguished from the Phlegm Dampness Pattern by teeth marked tongue, and from the deficiency patterns by pale tongue and thin pulse. Some other variables considered as factors of the Fire Heat Pattern such as tongue sore, thirst, vexing heat in the extremities, reddish yellow urine, yellow fur, constipation, rapid pulse, and flooding pulse did not register statistical significance. These results might be because those variables could be related to more than two patterns at once. For example, constipation can be seen in both the Fire Heat Pattern and the Qi Deficiency Pattern.

In the Phlegm Dampness Pattern, which is characterized by its impediment to Qi movement and its turbidity, heaviness, stickiness, and downward flowing properties [17], the following predictive logistic equation was derived log   (*P*/1 − *P*) = 0.578*(Overweight) − 0.754*(Fatigue) − 1.754*(Pale complexion) − 2.189*(Reddened complexion) − 2.719*(Flushed cheeks) + 1.496*(Pale tongue) + 2.365*(Slippery pulse) − 1.136. Slippery pulse was the most potent factor for determining the Phelgm Dampness Pattern, followed by pale tongue and overweight with the negatively quantified variables, the Phlegm Dampness Pattern could be distinguished from the Yin Deficiency Pattern by flushed cheeks, and from the Qi Deficiency Pattern by fatigue and pale complexion.

Qi is the basic element that constitutes the cosmos and, through its movement, changes and transformations. In the field of medicine, Qi refers both to the refined nutritive substance that flows within the human body as well as to its functional activities. Qi Deficiency generally leads to decreased visceral functions and lowered body resistance [17]. The predictive equation for the Qi Deficiency Pattern was log (*P*/1 − *P*) = −0.882*(Overweight) + 2.417*(Pale complexion) − 2.869*(Reddened complexion) − 2.252*(Flushed cheeks) + 1.451*(eyeball dryness) − 1.577*(Night sweating) − 1.474*(Nausea) + 1.165*(Reversal cold of the extremities − 2.100*(Thick fur) + 0.783*(Deep pulse) − 2.214*(Rapid pulse) + 0.993*(Vacuous pulse) − 2.572*(Slippery pulse) − 0.907. In this pattern, there were more negatively quantified variables than positively quantified variables. Thus, the exclusion of the other patterns was important. Overweight, thick fur, and slippery pulse were for the Phlegm Dampness Pattern, while reddened complexion and rapid pulse were for the Fire Heat Pattern.

Yin Deficiency indicates a pathologic change marked by deficiency of Yin with diminished moistening, calming, downbearing, and Yang-inhibiting function, leading to relative hyperactivity of Yang Qi [17]. The equation for the Yin Deficiency Pattern was log (*P*/1 − *P*) = 3.552*(Flushed cheeks) + 1.024*(Thirst) + 1.740*(Afternoon tidal fever) + 0.963*(Dry fur) + 0.982*(Rapid pulse) − 1.932*(Strong pulse) − 3.705. The variables, which are traditionally believed to be the indicators of the Yin Deficiency Pattern, were included in the equation, and these include flush cheeks, thirst, afternoon tidal fever, dry fur, and rapid pulse. However, thin pulse and heat in the palms and soles were not included, which might have been due to the relatively low frequency of such variables in the study data.

The finding that Korean OM doctors take tongue and pulse diagnosis seriously into their consideration is notable because all the patterns in this study basically included tongue and pulse diagnosis in their final equations. The Fire Heat Pattern and the Yin Deficiency Pattern have more positively quantified variables than negatively quantified variables. Thus, if some symptoms or signs reflect heat, the pattern could be determined easily. The Phlegm Dampness Pattern has two simple criteria overweight and slippery pulse. The Qi Deficiency Pattern is an exclusive pattern composed of many negatively quantified variables. This pattern could be identified merely on the condition that the other patterns were excluded. 

Recently, Kim et al. suggested a questionnaire for the Heat Pattern [7]. Among the eight variables in Kim's questionnaire, only one variable could be found in the equation in this study aversion to heat. The other seven variables including chest discomfort, constipation, and thirst were assessed and used in this study, but they were not significant. Park et al. also suggested a questionnaire composed of 14 variables for the Phlegm Dampness Pattern [10]. None of the variables could be found in the final equation for the Phlegm Dampness Pattern in this study. These differences between earlier studies and the present study might be attributed to the difference in methodologies used to select the variables. Kim's and Park's questionnaires were developed using the *Delphi* method from OM doctors. They set the variables by asking OM doctors what factors they think of most in identifying pattern. Thus, their variables were mere repetitions of theories, without practicality. The questionnaires did not offer anything new. 

With the results of this study, predictive logistic equations were suggested for four patterns the Fire Heat Pattern, the Phlegm Dampness Pattern, the Qi Deficiency Pattern, and the Yin Deficiency Pattern. These criteria would be helpful in determining the pattern of each stroke patient in clinics. However, further studies using large samples are necessary to validate and confirm these criteria.

## Figures and Tables

**Figure 1:**
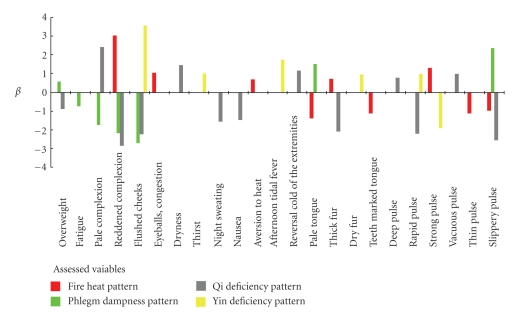
*β* regression coefficients of the variables in each of the patterns.

**Table 1:** Variables related to pattern-identification.

*Overweight*	Body Mass Index >23 (kg/m^2^)
*Insomnia*	Inability to sleep or abnormal wakefulness
*Fatigue*	Lack of strength
*Pale complexion*	A white complexion with a hint of blue or gray, oftern caused by yang collapse or exuberance of cold
*Yellow complexion*	Yellow discoloration of the face, generally suggesting accumulation of dampness
*Reddened complexion*	A complexion redder than normal, indicating the presence of heat
*Darkish complexion*	Dark discoloration of the face, often occurring in cold syndrome, water retention, or blood stasis
*Flushed cheeks*	Localized flush in the cheeks, indicating yin deficiency
*Headache*	Pain in the head
*Eye congestion*	Congestion in eyeballs indicating presence of heat
*Eyeball dryness*	Subjective feeling of dryness in the eyeballs
*Phlegm rale*	An abnormal breathing sound by phlegm in the airways
*Faint low voice*	A voice that is faint and low, scarcely audible
*Tongue sore*	Ulceration in the oral cavity or tongue
*Halitosis*	Bad smell from the mouth
*Thirst*	Feeling of dryness of the mouth with a desire to drink
*Bitter taste in the mouth*	A subjective bitter sensation in the mouth
*Profuse sweating*	Excessive sweating not related to a hot environment, physical exertion, or taking diaphoretics
*Night sweating*	Sweating during sleep that ceases on awakening
*Chest discomfort*	Unwell feeling of stuffiness and fullness in the chest
*Nausea*	An unpleasant sensation with an urge to vomit
*Borborigmus*	A rumbling sound made by the movement of gas in the intestines
*Aversion to heat*	Strong dislike of heat, also known as heat intolerance
*Afternoon tidal fever*	Fever more marked in the afternoon
*Heat in the palms and soles*	Subjective feverish feeling in the palms and soles
*Vexing heat in the extremities*	Uncomfortable heat sensation in the extremities
*Reversal cold of the extremities*	Pronounced cold in the extremities up to the knees and elbows, also the same as cold extremities
*Frequent urination*	Increased frequency of urination
*Reddish yellow urine*	Dark yellow or even reddish urine, indicating heat
*Constipation*	Hardened feces difficult to evacuate
*Pale tongue*	A tongue less red than normal, indicating Qi and blood deficiency
*Pale red tongue*	A tongue of normal color
*Red tongue*	A tongue redder than normal, indicating the presence of heat
*Bluish purple tongue*	A cyanotic tongue, indicating blood stasis or heat
*White fur*	A tongue coating white in color
*Yellow fur*	A tongue coating yellow in color
*Thick fur*	A tongue coating where the underlying tongue surface is not visible
*Dry fur*	A tongue coating that looks dry and feels dry to the touch
*Teeth marked tongue*	A tongue with dental indentations on its margin
*Enlarged tongue*	A tongue that is larger than normal, pale in color and delicate
*Spotted tongue*	A tongue with red, white or black spots
*Mirror tongue*	A completely smooth tongue free of coating, like a mirror
*Floating pulse*	A superficially located pulse which can be felt by light touch and grows faint on hard pressure
*Deep pulse*	A deeply located pulse which can only be felt when pressing hard
*Slow pulse*	Bradycardia
*Rapid pulse*	Tachycardia
*Strong pulse*	A general term for strongly beating pulse
*Vacuous pulse*	A general term for a feeble and void pulse
*Thin pulse*	A pulse as thin as a silk thread, straight and soft, feeble yet always perceptible upon hard pressure
*Slippery pulse*	A pulse coming and going smoothly like beads rolling on a plate
*Rough pulse*	A pulse coming and going unsmoothly with small, fine, slow joggling tempo like scraping bamboo with a knife
*Flooding pulse*	A pulse beating like dashing waves with forceful rising and gradual decline

**Table 2:** Demographic data of each pattern-identification.

	Fire heat	Phlegm dampness	Blood stasis	Qi deficiency	Yin deficiency
Total number	100	210	9	110	51
Age, yr	64.8 ± 11.1	68.3 ± 36.0	73.7 ± 10.4	65.5 ± 12.9	66.0 ± 14.7
Gender, male	77 (77.0)	105 (50.)	5 (55.6)	46 (41.8)	21 (41.2)
BMI (kg/m^2^)	24.2 ± 2.6	24.7 ± 2.9	22.6 ± 2.2	22.8 ± 2.8	22.5 ± 3.2
W/H ratio	0.95 ± 0.06	0.94 ± 0.05	0.95 ± 0.09	0.92 ± 0.07	0.91 ± 0.07
Ischemic stroke	82 (82.0)	190 (90.5)	8 (88.9)	91 (82.7)	42 (82.4)
TOAST*					
LAA	13 (13.0)	37 (17.6)	1 (11.1)	13 (11.8)	6 (11.8)
CE	5 (5.0)	16 (7.6)	0	9 (8.2)	6 (11.8)
SVO	64 (64.0)	135 (64.3)	5 (55.6)	66 (60.0)	29 (56.9)
SOE	0	2 (1.0)	1 (11.1)	1 (0.9)	1 (2.0)
SUE	0	0	1 (11.1)	2 (1.8)	0
Medical history					
Hypertension	55 (55.0)	150 (71.4)	5 (55.6)	64 (58.2)	24 (47.1)
Diabetic mellitus	24 (24.0)	73 (34.8)	3 (33.3)	23 (20.9)	11 (21.6)
Hyperlipidemia	7 (7.0)	45 (21.4)	0	17 (15.5)	3 (5.9)
Ischemic heart	7 (7.0)	18 (8.6)	1 (11.1)	11 (10.0)	8 (15.7)
Atrial fibrillation	10 (10.0)	13 (6.2)	1 (11.1)	7 (6.4)	6 (11.8)
Smoking	38 (38.0)	39 (18.6)	0	20 (18.2)	13 (25.5)

Values in parenthesis refer to %.

*TOAST classification includes LAA (large artery atherosclerosis), CE (cardiogenic embolism), SVO (small vessel occlusion), SOE (stroke of other determined etiology), and SUE (stroke of undetermined etiology).
